# Mitochondrial Dysfunction in Gulf War Illness Revealed by 31Phosphorus Magnetic Resonance Spectroscopy: A Case-Control Study

**DOI:** 10.1371/journal.pone.0092887

**Published:** 2014-03-27

**Authors:** Hayley J. Koslik, Gavin Hamilton, Beatrice A. Golomb

**Affiliations:** 1 Department of Medicine, University of California San Diego, La Jolla, California, United States of America; 2 Department of Radiology, University of California San Diego, La Jolla, California, United States of America; 3 Department of Family and Preventive Medicine, University of California San Diego, La Jolla, California, United States of America; Northwestern University Feinberg School of Medicine, United States of America

## Abstract

**Background:**

Approximately 1/3 of 1990-1 Gulf War veterans developed chronic multisymptom health problems. Implicated exposures bear mechanisms that adversely affect mitochondria. Symptoms emphasize fatigue, cognition and muscle (brain and muscle are aerobically demanding); with protean additional domains affected, compatible with mitochondrial impairment. Recent evidence supports treatments targeting cell bioenergetics (coenzyme10) to benefit Gulf War illness symptoms. However, no evidence has directly documented mitochondrial or bioenergetic impairment in Gulf War illness.

**Objective:**

We sought to objectively assess for mitochondrial dysfunction, examining post-exercise phosphocreatine-recovery time constant (**PCr-R**) using ^31^Phosphorus Magnetic Resonance Spectroscopy (**^31^P-MRS**), in Gulf War veterans with Gulf War illness compared to matched healthy controls. PCr-R has been described as a “robust and practical” index of mitochondrial status.

**Design and Participants:**

Case-control study from 2012–2013. Fourteen community-dwelling Gulf War veterans and matched controls from the San Diego area comprised 7 men meeting CDC and Kansas criteria for Gulf War illness, and 7 non-deployed healthy controls matched 1∶1 to cases on age, sex, and ethnicity.

**Outcome Measure:**

Calf muscle phosphocreatine was evaluated by ^31^P-MRS at rest, through 5 minutes of foot pedal depression exercise, and in recovery, to assess PCr-R. Paired t-tests compared cases to matched controls.

**Results:**

PCr-R was significantly prolonged in Gulf War illness cases vs their matched controls: control values, mean±SD, 29.0±8.7 seconds; case values 46.1±18.0 seconds; difference 17.1±14.9 seconds; p = 0.023. PCr-R was longer for cases relative to their matched controls for all but one pair; moreover while values clustered under 31 seconds for all but one control, they exceeded 35 seconds (with a spread up to 70 seconds) for all but one case.

**Discussion:**

These data provide the first direct evidence supporting mitochondrial dysfunction in Gulf War illness. Findings merit replication in a larger study and/or corroboration with additional mitochondrial assessment tools.

## Introduction

Of the ∼700,000 US troops deployed to the 1990-1 Persian Gulf theater, an estimated 175,000–250,000 (∼1/4–1/3 of those deployed), developed chronic multisymptom health problems often termed “Gulf War illness” (**GWI**) [1]. GWI is characterized by protean symptoms spanning multiple symptom “domains” (such as cognitive, fatigue, musculoskeletal). Gulf deployed veterans on average have more symptom domains affected, and greater severity and multiplicity of symptoms within domains, than Gulf era veterans who were not deployed [2]. Fatigue, exercise intolerance, cognitive difficulties, muscle pain and weakness, shortness of breath, gastrointestinal problems, sleep problems, behavior change, neurological findings, and skin problems are all elevated. GWI is defined by symptoms; a number of objective findings have been replicated, such as autonomic dysfunction, increased autoantibodies, reduced natural killer cell activity, and increased coagulation activation among others [3]. Studies generally show that affected veterans have not improved with time [4], [5], [6]; Gulf War veterans (**GWV**) continue to experience symptoms and impaired function 23 years later.

GWI is not equivalent to signature conditions of subsequent deployments to the region, such as posttraumatic stress disorder and traumatic brain injury. Indeed, stress and combat are demonstrably not the cause. While combat stress bears a dose-response relation to posttraumatic stress disorder (including in GWV); it bears no significant relationship to GWI in studies that adjust for other exposures [4]. Environmental factors are clearly inculpated in GWI. Many exposures were new, unique or excessive in the Gulf War. These include heat, sand, depleted uranium tanks/munitions, chemical agent resistant coating paint, and oil fires; as well as protections such as high numbers of multiple vaccines, anthrax vaccine, pyridostigmine bromide nerve agent pretreatment pills, pesticides and insect repellents, and permethrin-impregnated uniforms among others. Evidence most strongly implicates acetylcholinesterase inhibitor (**AChEi**) related exposures (which adhere to Hill's criteria for causality, include a dose-response relationship, and are buttressed by gene-exposure interaction data) [7]. Multiple vaccinations and anthrax vaccine also show relatively consistent epidemiological associations, but do not have the triangulating evidence for causality. AChEi related exposures include (carbamate) pyridostigmine bromide nerve agent pretreatment pills, given to an estimated 250,000 US troops [8]; carbamate and organophosphate pesticides [9], used aggressively and sometimes excessively to protect against insect vectors of disease [10], [11]; and organophosphate nerve gas, to which the Department of Defense estimates as many as ∼100,000 US troops were exposed in association with the demolition of the Khamisiyah munitions depot [12], with other possible nerve agent exposures [13]. Number of exposures experienced has also been linked to illness [14]; and exposure interactions, conceptually and empirically, may produce more problems [15].

**Figure 1:**
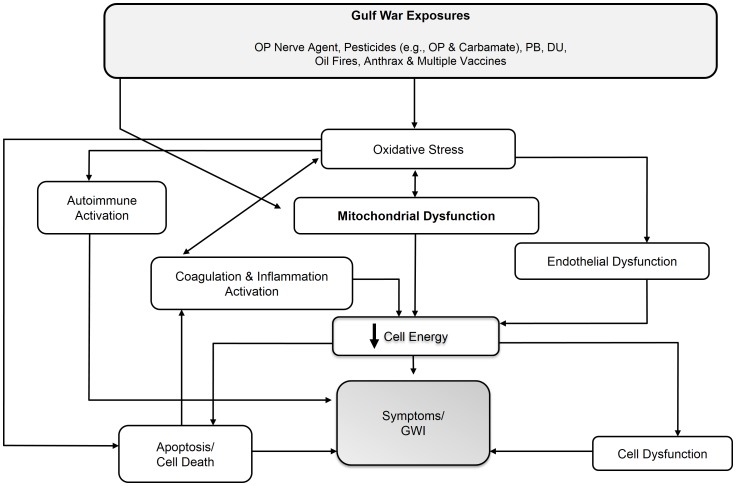
Hypothesized pathway (abridged version). DU = depleted uranium, GWI = Gulf War illness, OP = organophosphate, PB = pyridostigmine bromide.

The involvement of AChEi provides important information regarding potential mechanisms. Whereas AChEi toxicity is often viewed in terms of acetylcholinesterase inhibition, evidence shows that toxicity and lethality in fact relate decisively to (intertwined) oxidative stress (**OS**) and mitochondrial dysfunction (**MD**) [16] (phenomena that are tightly intertwined because the mitochondria are a leading target and source of reactive oxygen species [17], [18], [19]): indeed, animal evidence shows that high quality antioxidants administered just before or just after organophosphate pesticide exposure protect against lethality and chronic sequelae [20], [21]. We have shown that a mechanism involving OS-MD would explain the symptom profile (including which symptoms dominate – fatigue as well as central nervous system and muscle symptoms dominate in MD), symptom multiplicity, protean symptom character, variable latency to onset of symptoms, and the objective markers linked to GWI [3]. This mechanism would also provide for a subsidiary role for multiple other exposures for which mechanisms of action are classically considered to be unrelated, but which share in common induction of OS – potentiating MD and further OS. **Figure 1** depicts the mechanism we propose.

We sought to directly assess the hypothesis of MD in a pilot study of ill GWVs vs matched controls, using ^31^Phosphorus Magnetic Resonance Spectroscopy (**^31^P-MRS**) to measure post-exercise phosphocreatine-recovery time constant (**PCr-R**), a marker that serves as “an estimate of net oxidative ATP synthesis,” [22] and “a robust and practical way to study mitochondrial regulation and to quantify effective mitochondrial defects in vivo” [23]. We also assessed resting brain and muscle phosphocreatine (**PCr**).

## Methods

### Ethics statement

The study protocol was approved by the University of California, San Diego Human Research Protections Program. All subjects gave written informed consent.

### Participants

GWI cases comprised 7 veterans who were deployed to the Persian Gulf theater between August 1990 and July 1991, and met Centers for Disease Control and Prevention (**CDC**) and Kansas criteria for GWI. CDC criteria require presence of one or more symptoms in each of at least 2 of the 3 domains of fatigue, musculoskeletal, and mood-cognitive. Kansas GWI criteria require that veterans have multiple symptoms within a qualifying domain, and/or symptom(s) of at least moderate severity, in at least 3 of the 6 domains of neurological-cognitive-mood, fatigue/sleep problems, respiratory, pain, gastrointestinal, and skin [2]. To qualify for either, symptoms must have been present for at least 6 months and not present prior to the Gulf conflict.

Controls were 7 non-deployed individuals matched 1∶1 to GWI cases on sex, age (within 38 months) and ethnicity. To qualify, controls must meet neither CDC nor Kansas symptom criteria for GWI, nor Kansas exclusionary criteria (concurrent significant conditions such as diabetes, heart disease, cancer, that could produce symptoms that might be confused for GWI), nor could any inquired-about symptom be greater than mild in severity.

All prospective participants were screened prior to enrollment and again on the day of their visit to ensure ^31^P-MRS eligibility. Requirements included no metal that might pose a problem with magnetic resonance assessment (e.g. no prosthetic devices, shrapnel, welding as a hobby); and weight ≤300 lbs.

### ^31^P-MRS (performed by Dr. Hamilton)

^31^P-MR spectra were acquired on a 3 Tesla GE Signa EXCITE HD scanner (GE Healthcare, Waukesha, WI). The 1H signal was acquired using the body coil for collection of multiplanar localization images and for shimming. Participants were scanned in the supine position. The ^31^P-MR spectra were collected with a 5-inch diameter surface coil, using a slice selective free induction decay sequence with a repetition time of 3 seconds. Spectra had a sampling interval of 0.2 ms; 2048 data points were collected. For muscle spectra, the coil was placed under the calf. The free induction decay sequence excited a thick slice (60 mm) parallel to the coil. The slice was positioned close to the coil to maximize signal-to-noise whilst attempting to minimize inclusion of surface features in the region close to the coil. The intrinsic weighting of the coil provided the remaining localization. For the exercise protocol, a spectrum was collected every 3 seconds during 2 minutes of rest (providing resting muscle spectra), then 5 minutes of exercise (repetitively depressing, as far as they were able, a metal-free pedal, similar to depressing a car pedal, with elastic bands providing resistance). This was followed by 6 minutes of recovery. A velcro strap across subjects' thighs limited extraneous movement during exercise.

Brain spectra were collected at rest with 128 signal averages, placing the coil at the front and back of the head, successively. A clear plexiglass head box with 3 sides reduced motion artifact; foam pads were placed for comfort and neck support on the MR table.

Headphones with a microphone were given to every participant to dampen noise generated from the MR scans, and to allow communication with study staff and MR technicians. Participants selected a radio station to listen to during the scans.

### ^31^P-MRS analysis

The raw spectra files were transferred and analyzed off-line. All spectroscopy analyses were carried out by a single observer (Dr. Hamilton, blinded to case-control status) who monitored spectral quality during the analysis process, to confirm quality of acquisition. The inherently low signal-to-noise of ^31^P-MR spectra and the complex overlapping peak structure of the spectra were addressed via AMARES algorithm [24] included in the MRUI software program [25], using prior knowledge adapted from an approach previously used by Dr. Hamilton at 1.5T [26]. For the resting spectra (only), signal to noise was adequate to permit truncation of the first 2 ms of the signal to remove the broad component to the residual, which is the difference between the full signal and the fit of the truncated signal. This broad component corresponds to signals from motion-restricted phospholipid in cell membrane and vesicle bilayers [27]. The post-exercise PCr-R time constant was procured, as relative change in the area. For the rest spectra, the areas of the PCr peak were expressed as a fraction of the total ^31^P-MRS signal (sum of the areas of all visible peaks); intracellular pH values were determined [28], [29].

### PCr-R analysis

Paired t-tests compared values of PCr-R (and of secondary measures) in cases relative to their matched controls. Two-sided p-values <0.05 designated statistical significance. Analyses were conducted using Stata version 8.0.

## Results

Subjects (cases and controls) were male, with mean age 53.9 (range 44 to 65 years). 85.7% were Caucasian, 14.3% African American.

**Figure 2:**
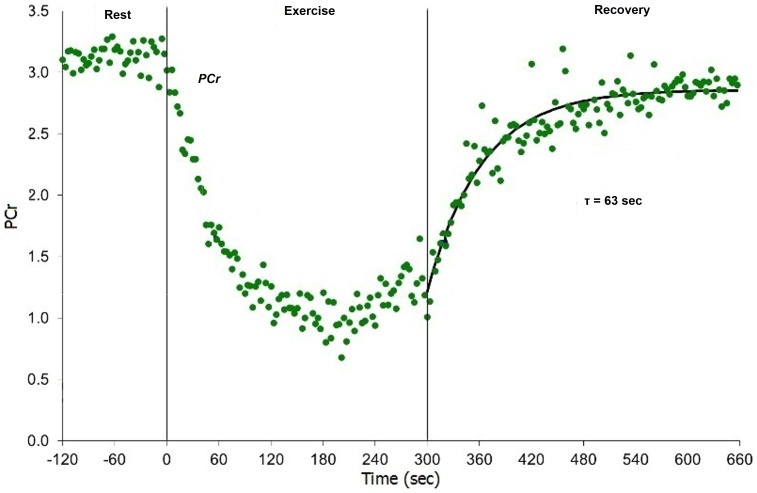
PCr-R rest-exercise-recovery profile for a GWI case. GWI  =  Gulf War illness; PCr  =  phosphocreatine; PCr-R  =  phosphocreatine-recovery post-exercise. Rest-exercise-recovery phosphocreatine response curve for a Gulf War veteran (rest −120–0 sec, exercise 0–300 sec, recovery 300–660 sec) (Case 5).

All participants performed the exercise rest/recovery task and provided PCr-R data. **Figure 2** shows an example PCr rest-exercise-recovery curve. Resting PCr and pH values did not significantly differ in cases vs controls.

**Figure 3:**
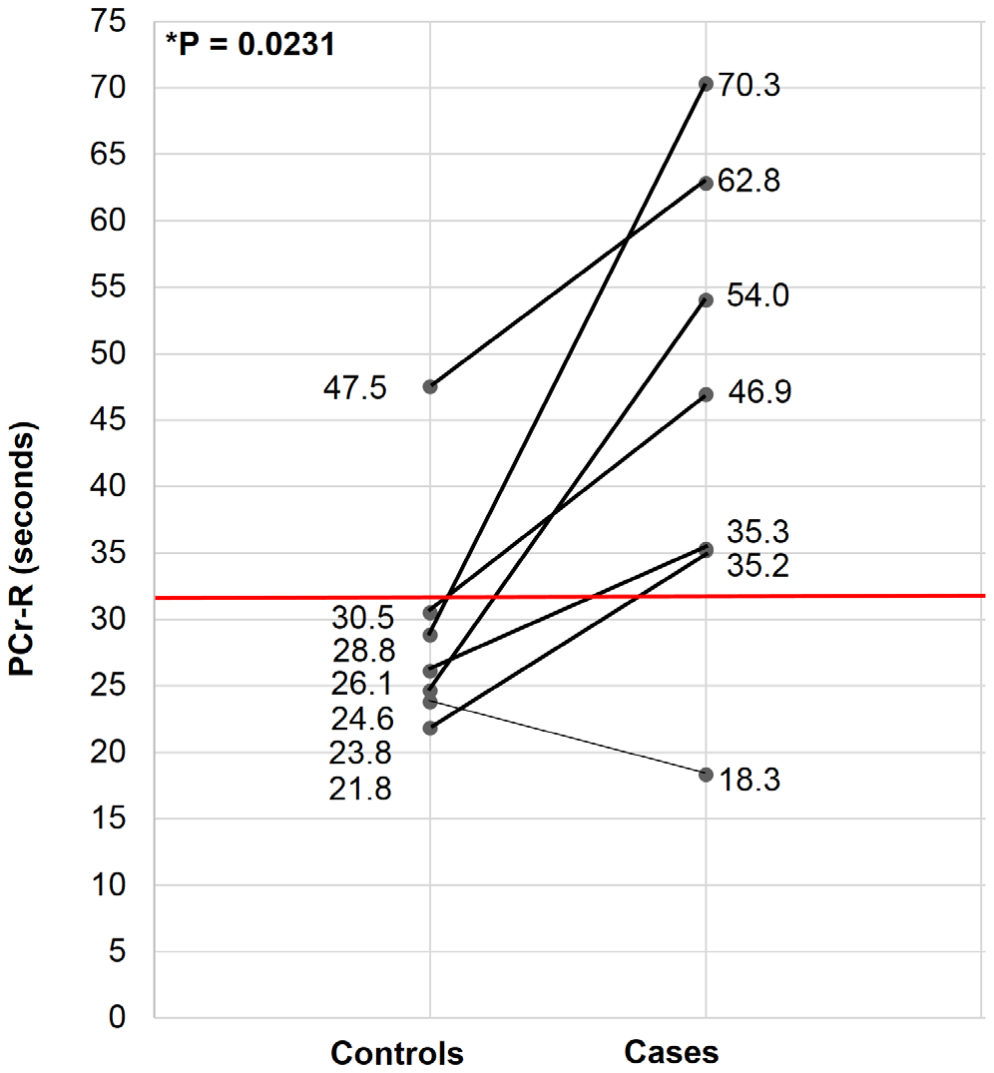
PCr-R of GWV vs Matched Controls. GWV  =  Gulf War veteran; PCr-R  =  phosphocreatine-recovery post-exercise. Line segments connect controls to their case matches. Note clustering of most control (but not case) PCr-R values under 31 seconds, with widely distributed case values primarily in excess of 35 seconds.

**Figure 3** **and** **Table 1** show PCr-R findings. Veterans with GWI had significantly prolonged PCr-R relative to their matched healthy controls (p = 0.023). For 6 of the 7 pairs, PCr-R was greater in the GWI case than in their matched healthy control. Moreover, with only one exception, control participants all had PCr-R values clustered under 31 seconds; while in contrast, all but one case had values distributed over 35 seconds, up to a maximum of ∼70 seconds.

**Table 1:** PCr-R by ^31^P-MRS in GWI Cases vs Matched Controls.

	Case PCr-R (sec)	Difference	Control PCr-R (matched to that case) (sec)	Difference (sec)
Pair 1	35.200	>	21.800	+13.4
Pair 2	70.300	>	28.800	+41.2
Pair 3	54.000	>	24.600	+29.4
Pair 4	18.300	<	23.800	−5.5
Pair 5	62.800	>	47.500	+15.3
Pair 6	35.300	>	26.100	+9.2
Pair 7	46.900	>	30.500	+16.4

GWI  =  Gulf War illness; PCr-R  =  phosphocreatine-recovery post-exercise; ^31^P-MRS  =  ^31^Phosphorus Magnetic Resonance Spectroscopy; sec  =  seconds.

The case that provided the exception had the lowest PCr-R value of all subjects – cases or controls. Of note, whereas all other participants with GWI cited their pre-Gulf health as “excellent” or “very good”, this was the sole case to rate their health prior to their Gulf War participation as merely “good,” perhaps suggesting an unrelated prior health condition.

## Discussion

To our knowledge, this is the first study to document prolongation of PCr-R in GWI, or indeed to assess PCr-R or MD in GWI. PCr-R is viewed as a “robust” index of MD [23]; thus, this study supports the hypothesis that mitochondrial pathology is present in GWI. Not only did all-but-one matched case-control pair show the hypothesized directionality, but values for all controls except one were clustered under 31 seconds, while values for all cases but one were distributed over 35 seconds, and up to 70 seconds, providing relatively striking separation. Lower pre-Gulf health was reported by the PCr-R-nonconforming veteran, relative to all other assessed veterans, affording the prospect that different health factors may have been at play in that individual.

These findings add empirical support to evidence previously assembled, making a case for a role for MD (and interrelated OS, which promotes and is caused by MD [17], [18]) in GWI [3].They also fit with evidence suggesting benefit of coenzyme Q10 for symptoms in GWI [30]. MD is *expected* based on known exposure relations to GWI [3], [16], [31]; and indeed, fully accounts for key “perplexing” clinical features of GWI not equally accounted for by any proposed alternative hypothesis, including which symptoms dominate (fatigue, muscle and brain, the latter two because they are energetically demanding postmitotic tissues), the spectrum of other symptoms less consistently present, the high symptom multiplicity, protean character, and variable latency to onset [3]. A role for MD also coheres with evidence of amyotrophic lateral sclerosis, observed in several studies to be present at elevated rates among GWV [32], [33], [34]: this condition involves MD, in a vicious cycle with OS [35].

Physiological dysfunction in GWI made evident following exercise has been reported for gene expression and imaging parameters [36], [37], [38]. Our finding of prolongation of post-exercise PCr-R represents a further instance in which exercise unmasks altered physiology in GWI. Of note, an oxidative-mitochondrial foundation could be postulated to underlie the other observed exercise-related findings.

This study has limitations. Most importantly, the sample size was small. However significance of the difference between cases and matched controls in the face of a small sample requires a correspondingly large effect size. Additionally, close matching of cases with their controls on key parameters advantages authority of the comparison. So too does the relatively striking separation between PCr-R values for the case and control groups. Exercise and diet patterns of controls were not elicited. Some otherwise eligible GWV with GWI were excluded from participation for MR safety reasons, due to historical or current hobbies involving welding, soldering, or grinding (associated with risk of unrecognized embedded metal around the eyes). However, this exclusion applied equally to cases and controls.

In summary, veterans of the 1990-1 Persian Gulf War who are affected by GWI show prolonged post-exercise recovery of PCr on ^31^P-MRS, consistent with a role for MD in GWI. ^31^P-MRS may provide an objective diagnostic tool for GWI that can be tracked with treatment. Findings of this study require replication and ideally assessment in a larger sample, and will benefit from triangulating validation with additional mitochondrial assessment tools.

## References

[1] Binns JH, Cherry N, Golomb BA, Graves JC, Haley RW, et al. (2004) Research Advisory Committee on Gulf War Veterans' Illnesses: Scientific Progress in Understanding Gulf War Veterans' Illnesses: Report and Recommendations. Washington, D.C.

[2] Steele L (2000) Prevalence and patterns of Gulf War illness in Kansas veterans: association of symptoms with characteristics of person, place, and time of military service. Am J Epidemiol 152: 992–1002. 11092441 10.1093/aje/152.10.992

[3] Golomb BA (2012) Oxidative Stress and Mitochondrial Injury in Chronic Multisymptom Conditions: From Gulf War Illness to Autism Spectrum Disorder. Available: http://precedings.nature.com/documents/6847/version/1. Accessed 2014 Mar 3.

[4] Binns JH, Barlow C, Bloom FE, Clauw DJ, Golomb BA, et al. (2008) Gulf War Illness and the Health of Gulf War Veterans. Scientific Findings and Recommendations. Washington D.C.: U.S. Government Printing Office.

[5] Ozakinci G, Hallman WK, Kipen HM (2006) Persistence of symptoms in veterans of the First Gulf War: 5-year follow-up. Environ Health Perspect 114: 1553–1557. 17035142 10.1289/ehp.9251 PMC1626433

[6] Hotopf M, David AS, Hull L, Nikalaou V, Unwin C, et al (2003) Gulf war illness–better, worse, or just the same? A cohort study. BMJ 327: 1370. 14670878 10.1136/bmj.327.7428.1370 PMC292982

[7] Golomb BA (2008) Acetylcholinesterase inhibitors and Gulf War illnesses. Proc Natl Acad Sci U S A 105: 4295–4300. 18332428 10.1073/pnas.0711986105 PMC2393741

[8] Golomb BA (1999) A Review of the Scientific Literature as it Pertains to Gulf War Illnesses, Vol 2: Pyridostigmine Bromide. Washington, DC: RAND. 385 p.

[9] Cecchine G, Golomb BA, Hilborne LH, Spektor DM, Anthony CR (2000) A Review of the Scientific Literature as it Pertains to Gulf War Illnesses, Vol 8: Pesticides. Santa Monica, CA: RAND. 182 p.

[10] Department of Defense (2003) Environmental Exposure Report. Pesticides. Final Report. Available: http://www.gulflink.osd.mil/pest_final/index.html. Accessed 2014 Mar 3.

[11] Fricker RD, Reardon E, Spektor DM, Cotton SK, Hawes-Dawson J, et al. (2000) A Review of the Scientific Literature as It Pertains to Gulf War Illnesses. Volume 12: Pesticide Use During the Gulf War: A Survey of Gulf War Veterans. Santa Monica, CA: RAND, MR-1018/12-OSD.

[12] Gillert DJ (1997) DoD Says 98,910 Exposed to Low Levels of Nerve Agent. Available: http://www.defense.gov/News/NewsArticle.aspx?ID=41500. Accessed 2014 Mar 6.

[13] General Accounting Office (2004) Gulf War Illnesses: DOD's Conclusions about U.S. Troops' Exposure Cannot Be Adequately Supported. GAO report number GAO-04-159. Available: http://www.gao.gov/new.items/d04159.pdf. Accessed 2014 Mar 3.

[14] Kroenke K, Koslowe P, Roy M (1998) Symptoms in 18,495 Persian Gulf War veterans. Latency of onset and lack of association with self-reported exposures. J Occup Environ Med 40: 520–528. 9636932 10.1097/00043764-199806000-00004

[15] Abou-Donia MB, Wilmarth KR, Abdel-Rahman AA, Jensen KF, Oehme FW, et al (1996) Increased neurotoxicity following concurrent exposure to pyridostigmine bromide, DEET, and chlorpyrifos. Fundam Appl Toxicol 34: 201–222. 8954750 10.1006/faat.1996.0190

[16] Milatovic D, Gupta RC, Aschner M (2006) Anticholinesterase toxicity and oxidative stress. ScientificWorldJournal 6: 295–310. 16518518 10.1100/tsw.2006.38 PMC5917369

[17] Lee HC, Wei YH (1997) Role of Mitochondria in Human Aging. J Biomed Sci 4: 319–326. 12386380 10.1007/BF02258357

[18] Genova ML, Pich MM, Bernacchia A, Bianchi C, Biondi A, et al (2004) The mitochondrial production of reactive oxygen species in relation to aging and pathology. Ann N Y Acad Sci 1011: 86–100. 15126287 10.1007/978-3-662-41088-2_10

[19] Wei YH (1998) Oxidative stress and mitochondrial DNA mutations in human aging. Proc Soc Exp Biol Med 217: 53–63. 9421207 10.3181/00379727-217-44205

[20] Pena-Llopis S, Ferrando MD, Pena JB (2003) Fish tolerance to organophosphate-induced oxidative stress is dependent on the glutathione metabolism and enhanced by N-acetylcysteine. Aquat Toxicol 65: 337–360. 14568351 10.1016/s0166-445x(03)00148-6

[21] Pena-Llopis S, Ferrando MD, Pena JB (2003) Increased recovery of brain acetylcholinesterase activity in dichlorvos-intoxicated European eels Anguilla anguilla by bath treatment with N-acetylcysteine. Dis Aquat Organ 55: 237–245. 13677510 10.3354/dao055237

[22] Kemp GJ, Taylor DJ, Radda GK (1993) Control of phosphocreatine resynthesis during recovery from exercise in human skeletal muscle. NMR Biomed 6: 66–72. 8457428 10.1002/nbm.1940060111

[23] Thompson CH, Kemp GJ, Sanderson AL, Radda GK (1995) Skeletal muscle mitochondrial function studied by kinetic analysis of postexercise phosphocreatine resynthesis. J Appl Physiol 78: 2131–2139. 7665409 10.1152/jappl.1995.78.6.2131

[24] Vanhamme L, van den Boogaart A, Van Huffel S (1997) Improved method for accurate and efficient quantification of MRS data with use of prior knowledge. J Magn Reson 129: 35–43. 9405214 10.1006/jmre.1997.1244

[25] Naressi A, Couturier C, Castang I, de Beer R, Graveron-Demilly D (2001) Java-based graphical user interface for MRUI, a software package for quantitation of in vivo/medical magnetic resonance spectroscopy signals. Comput Biol Med 31: 269–286. 11334636 10.1016/s0010-4825(01)00006-3

[26] Hamilton G, Patel N, Forton DM, Hajnal JV, Taylor-Robinson SD (2003) Prior knowledge for time domain quantification of in vivo brain or liver 31P MR spectra. NMR Biomed 16: 168–176. 12884361 10.1002/nbm.821

[27] Murphy EJ, Rajagopalan B, Brindle KM, Radda GK (1989) Phospholipid bilayer contribution to 31P NMR spectra in vivo. Magn Reson Med 12: 282–289. 2559292 10.1002/mrm.1910120218

[28] Golomb BA, Erickson LC, Scott-Van Zeeland AA, Koperski S, Haas RH, et al. (2013) Assessing Bioenergetic Compromise in Autism Spectrum Disorder With 31P Magnetic Resonance Spectroscopy: Preliminary Report. J Child Neurol. 10.1177/0883073813498466 PMC3931549 24141271

[29] Hamilton G, Allsop JM, Patel N, Forton DM, Thomas HC, et al (2006) Variations due to analysis technique in intracellular pH measurements in simulated and in vivo 31P MR spectra of the human brain. J Magn Reson Imaging 23: 459–464. 16506142 10.1002/jmri.20524

[30] Golomb BA, Koperski S, Rock CL, Devaraj S, Allison M, et al. (2014) Coenzyme Q10 Benefits Symptoms in Gulf War Veterans: Results of a Randomized Double-Blind Pilot Study. Neural Comput, in press. 10.1162/NECO_a_00659 25149705

[31] Chan JY, Chan SH, Dai KY, Cheng HL, Chou JL, et al (2006) Cholinergic-receptor-independent dysfunction of mitochondrial respiratory chain enzymes, reduced mitochondrial transmembrane potential and ATP depletion underlie necrotic cell death induced by the organophosphate poison mevinphos. Neuropharmacology 51: 1109–1119. 16984802 10.1016/j.neuropharm.2006.06.024

[32] Coffman CJ, Horner RD, Grambow SC, Lindquist J (2005) Estimating the occurrence of amyotrophic lateral sclerosis among Gulf War (1990-1991) veterans using capture-recapture methods. Neuroepidemiology 24: 141–150. 15650320 10.1159/000083297

[33] Haley RW (2003) Excess incidence of ALS in young Gulf War veterans. Neurology 61: 750–756. 14504316 10.1212/wnl.61.6.750

[34] Horner RD, Kamins KG, Feussner JR, Grambow SC, Hoff-Lindquist J, et al (2003) Occurrence of amyotrophic lateral sclerosis among Gulf War veterans. Neurology 61: 742–749. 14504315 10.1212/01.wnl.0000069922.32557.ca

[35] Golomb BA, Kwon EK, Koperski S, Evans MA (2009) Amyotrophic lateral sclerosis-like conditions in possible association with cholesterol-lowering drugs: an analysis of patient reports to the University of California, San Diego (UCSD) Statin Effects Study. Drug Saf 32: 649–661. 19591530 10.2165/00002018-200932080-00004

[36] Whistler T, Fletcher M, Lonergan W, Zeng X-R, Lin J-M, et al (2009) Impaired immune function in Gulf War Illness. BMC Medical Genomics 2: 12. 19265525 10.1186/1755-8794-2-12 PMC2657162

[37] Broderick G, Ben-Hamo R, Vashishtha S, Efroni S, Nathanson L, et al (2013) Altered immune pathway activity under exercise challenge in Gulf War Illness: an exploratory analysis. Brain Behav Immun 28: 159–169. 23201588 10.1016/j.bbi.2012.11.007

[38] Rayhan RU, Stevens BW, Raksit MP, Ripple JA, Timbol CR, et al (2013) Exercise Challenge in Gulf War Illness Reveals Two Subgroups with Altered Brain Structure and Function. PLoS ONE 8: e63903. 23798990 10.1371/journal.pone.0063903 PMC3683000

